# Ethanol production improvement driven by genome-scale metabolic modeling and sensitivity analysis in *Scheffersomyces stipitis*

**DOI:** 10.1371/journal.pone.0180074

**Published:** 2017-06-28

**Authors:** Alejandro Acevedo, Raúl Conejeros, Germán Aroca

**Affiliations:** Escuela de Ingeniería Bioquímica, Pontificia Universidad Católica de Valparaíso, Av. Brasil 2085, Valparaíso, Chile; National Renewable Energy Laboratory, UNITED STATES

## Abstract

The yeast *Scheffersomyces stipitis* naturally produces ethanol from xylose, however reaching high ethanol yields is strongly dependent on aeration conditions. It has been reported that changes in the availability of NAD(H/^+^) cofactors can improve fermentation in some microorganisms. In this work genome-scale metabolic modeling and phenotypic phase plane analysis were used to characterize metabolic response on a range of uptake rates. Sensitivity analysis was used to assess the effect of ARC on ethanol production indicating that modifying ARC by inhibiting the respiratory chain ethanol production can be improved. It was shown experimentally in batch culture using Rotenone as an inhibitor of the mitochondrial NADH dehydrogenase complex I (CINADH), increasing ethanol yield by 18%. Furthermore, trajectories for uptakes rates, specific productivity and specific growth rate were determined by modeling the batch culture, to calculate ARC associated to the addition of CINADH inhibitor. Results showed that the increment in ethanol production via respiratory inhibition is due to excess in ARC, which generates an increase in ethanol production. Thus ethanol production improvement could be predicted by a change in ARC.

## Introduction

Xylose fermentation to ethanol is essential to achieve economically viable biofuel production from lignocellulosic biomass [1–5]. *Scheffersomyces stipitis* [6] is a xylose-fermenting yeast which has been extensively studied in recent years, research works include analyses based on genomics [7, 8], transcriptomics [9], proteomics [10] and genome-scale metabolic reconstructions [11, 12]. *S. stipitis* is Crabtree-negative [13, 14] which shows a strong dependency between ethanol production and oxygen supply regardless the carbon source availability or dilution rates [15–17]. In *S. stipitis*, the yield of ethanol from xylose decreases with high oxygen supply and increases at low oxygen supply, nonetheless, productivity could drop in the latter case [18, 19]. Hereafter, the yield of ethanol from xylose it will be referred as yield.

Also, at complete anaerobic conditions, *S. stipitis* is able to grow the equivalent of just one doubling time before growth and ethanol production stop [20, 21]. Culture conditions associated to high yield in *S. stipitis* are in a narrow range of oxygen concentrations, so that an effective control of oxygen supply is required [22–28]. *S. stipitis* has the proton translocating NADH dehydrogenase complex I (NDH1) and an alternative oxidase (AOX) in the respiratory chain, both absent in the Crabtree-positive yeast *Saccharomyces cerevisiae* [29]. This feature allows in *S. stipitis* a higher oxidative capacity for NADH at the respiratory chain than *S. cerevisiae* [30]. Fermentative metabolism is affected by NAD(H/^+^) cofactors, several reports indicate that cofactor manipulation is a useful tool to improve fermentation [31]. In this regard, it has been shown in *E. coli* that an increase in NADH availability induces fermentation by stimulating pathways which are normally inactive under aerobic conditions [32]. Besides, Vemuri *et al*. [33] were able to reduce the Crabtree-effect in a great extent by introducing a heterologous alternative oxidase (AOX) at the mitochondrion, which showed that NADH oxidative capacity is directly related to the occurrence of the Crabtree-effect in *S. cerevisiae*. Furthermore, Hou *et al*. [34] using a *S. cerevisiae* strain disabled for formate anabolism, and modified to overexpress the native NAD dependent formate dehydrogenase, were able to induce fermentation by increasing intracellular NADH concentration via formate supplementation.

Since xylose metabolism yields a positive NADH balance, cofactor usage has been shown to be important on ethanol production when xylose is used [35, 36]. *S. stipitis* may prevent NADH excess via its high NADH oxidative capacity at the respiratory chain and by converting NADH to NADPH through a bypass at the tricarboxylic acids cycle (TCA) [8, 11]. Moreover, *S. stipitis* may be able to use the arabinose assimilation pathway backwards, oxidizing NADH and producing polyols, in fact, polyol accumulation in *S. stipitis* is several times greater than in *S. cerevisiae*; 317 times for arabitol and 46 times for ribitol [37]. On the other hand, decreasing NADH oxidative capacity in *S. stipitis* by modifying enzymes of the respiratory chain increases yield even under aerobic conditions [30, 38, 39], suggesting that changes in the availability of the NAD(H/^+^) redox pair affect the dependency between ethanol production and oxygen supply. The different phenotypic phases of S. stipitis metabolism had been studied in an experimentally validated core metabolic model [36]. This study points out the fact that xylitol and acetic acid are not always produced. There is only one phenotypic phase where this metabolic this by-product can be produced.

An useful way to study metabolic behaviour is by analyzing the flux distribution in the stoichiometric network, the method most extensively used to this purpose is the Flux Balance Analysis (FBA) [40, 41]. Through a sensitivity analysis of the FBA it can be determined how a perturbation on the availability of a given metabolite affects the metabolic objective considered in the model [42, 43]. In the case of the sensitivity to the availability of reducing power supplied by redox cofactors, it is considered as the net effect of changes in the redox pair, that is the difference between NADH and NAD sensitivities. Edwards *et al*, [44] proposed this index calling it redox shadow price, in the present work it is referred as the sensitivity to the Available Reducing Capacity (ARC). This work proposes to improve the yield of ethanol from xylose by modifying the ARC in *S. stipitis*. The metabolic models were obtained from genome-scale metabolic reconstructions [45], Flux Balance Analysis (FBA) [40, 41] was used to determine metabolic states, Phenotypic Phase Plane (PhPP) analysis [44] was used to characterize the metabolic behaviour on a range of uptake rates, and to assess the sensitivity response of ethanol production to changes in ARC, the sensitivity analysis of the FBA was used. *In-silico* analyses made on *S. stipitis* were also performed on *S. cerevisiae*, which it is well-known for its ethanol production capacity. Also, deletions found *in-silico* by Acevedo *et al*, [46] in *S. stipitis* to improve ethanol production were analyzed using the above mentioned tools.

*In-silico* results indicated that inhibiting components of the respiratory chain modifies ARC and it allows to associate ethanol production to growth. Subsequently, *S. stipitis* batch cultures were performed adding respiratory inhibitors in order to improve yield. Batch culture essays showed that yield increases by 18% when inhibiting the mitochondrial NADH dehydrogenase complex I. Next, by using data from batch cultures and a kinetic model of the culture, uptakes rates were determined and were used as constraints in the FBA of *S. stipitis* in order to determine the ethanol sensitivities associated to batch cultures. Results showed that the observed increase in ethanol production via respiratory inhibition is related to an excess in ARC for biomass production and to a positive response of ethanol production to this excess.

## Materials and methods

### Models

The following genome scale metabolic reconstructions were used: for *S. cerevisiae* the IMM904 model [47] which has 1577 reactions and 1228 metabolites and for *S. stipitis* the IBB814 model [11] which has 1371 reactions and 971 metabolites. Both models had been experimentally validated in several researches [43, 48–53].

### Flux Balance Analysis (FBA)

To determine metabolic states FBA was used [40, 41]. Loop law constraints were added to all FBA calculations according to Schellenberg *et al*., [54] method so that infeasible loops were not allowed. Maximization of biomass production was considered as objective function since it has been useful to characterize metabolic phenotypes in yeast [11, 43, 47–49, 51–53].

The flux-carrying set (FCS) is defined as the all non-zero fluxes obtained from a FBA. The changes in the FCS are characterized by the fluxes which are increased, decreased, turned on or turned off due to a imposed modification in any given flux.

### Computational tools

Computational methods were performed using Matlab2012a as programming environment. FBA routines were carried out using COBRA (*Constraint-Based Reconstruction and Analysis*) Toolbox version 2.0 [55]. TomLab/CPLEX version 7.8 was used as solver. All Matlab codes for COBRA function can be found online at the website: http://opencobra.sourceforge.net/ .

### Phenotypic phase planes analysis

Phenotypic phase planes (PhPPs) analysis [44] was performed characterizing all optimal flux distributions as a function of carbon source and oxygen uptake rates. PhPPs were obtained by varying in a step wise fashion the two fluxes and solving the FBA.

### Sensitivity analysis

The sensitivity analysis consisted in determining the shadows prices associated to the dual problem of the FBA, which can be used to characterize the effect of changes in the availability of metabolites [42–44]. The sensitivity $\lambda_{i}^{Z}$ (Eq (1)) represents the response of the objective function *Z* to a perturbation on the availability of a metabolite *i*. Each sensitivity is defined as:

$$\begin{array}{c} \lambda_{i}^{Z}=-\frac{\partial Z}{\partial b_{i}^{r}} \end{array}$$ (1)

Where *b*_*i*_ corresponds to the mass balance for metabolite *i* and super index *r* denotes a relaxation in the steady state. The sensitivity corresponds to the Lagrange multipliers associated to the dual optimization problem. These values were obtained from the solution vector of the dual problem (vector *m* × 1 where m is the number of metabolites) in the COBRA function optimizeCbModel. From de Eq (1) it can be noted that if the response of *Z* to an increment in $b_{i}^{r}$ is an increase in its value, $\lambda_{i}^{Z}$ should be less than zero. On the other hand, if *Z* decreases $\lambda_{i}^{Z}$ should be greater than zero, and if *Z* does not change $\lambda_{i}^{Z}$ is equal to zero. In this sense, the sensitivity value can be interpreted as a state of resource availability showing if a given metabolite is limiting ($\lambda_{i}^{Z}<0$), in excess ($\lambda_{i}^{Z}>0$) or it has no effect on *Z* ($\lambda_{i}^{Z}$ = 0).

### Sensitivity to changes in the available reducing capacity supply the NAD(H/+) redox pair

As described by [44], the sensitivity of an metabolic objective *Z* to change in a redox pair such as NAD(H/+) is define as:

$$\begin{array}{c} \lambda_{ARC}^{Z}=\lambda_{NADH}^{Z}-\lambda_{NAD}^{Z} \end{array}$$ (2)

Where the difference between $\lambda_{NADH}^{Z}$ and $\lambda_{NAD}^{Z}$ represents the net effect of changes in both species. Then $\lambda_{ARC}^{Z}$ is an index of the response to changes in available reducing capacity supplied by the redox coupled, shortly referred as ARC.

### Determination of the sensitivity of ethanol production to ARC changes

The sensitivity of ethanol production to ARC changes $\lambda_{ARC}^{etoh}$ was calculated throughout the PhPPs. This was achieved by determining the PhPP using firstly the biomass production as objective function which allowed characterizing metabolic states with biological sense. Then all flux values associated to that state, including biomass production, were used as constraints an ethanol production was used as objective function. This procedure allowed obtaining the $\lambda_{ARC}^{etoh}$ values associated to the PhPP.

### Computational methods for culture modeling

#### Culture model

*S. stipitis* batch culture was modeled using differential equations in order to represent biomass, xylose and ethanol mass balances. Thereby the culture model was defined as:

$$\begin{array}{c} \frac{dx}{dt}=x\cdot \mu \end{array}$$ (3)

$$\begin{array}{c} \frac{dS}{dt}=-q_{S}\cdot x \end{array}$$ (4)

$$\begin{array}{c} \frac{dP}{dt}=q_{P}\cdot x \end{array}$$ (5)

Where, *x* is biomass concentration (g⋅L^−1^). *S*: is xylose concentration (g⋅L^−1^) and *P* is ethanol concentration (g⋅L^−1^). Monod equation was used to describe specific growth as follows:

$$\begin{array}{c} \mu =\frac{\mu_{max}\cdot S}{S+K_{s}} \end{array}$$ (6)

Where *μ* is the specific growth rate (h^−1^), *S* is xylose concentration (g⋅L^−1^), *K*_*s*_ is the substrate saturation constant (g⋅L^−1^) and *μ*_*max*_ is the maximum specific growth rate. The specific uptake rate of xylose was determined based on substrate usage mass balance as follows:

$$\begin{array}{c} q_{S}=\frac{\mu }{Y_{o}}+m+\frac{q_{P}}{Y_{P}} \end{array}$$ (7)

Where, q_*S*_ is the specific uptake rate of xylose (g⋅gcel^−1^ h^−1^), *Y*_*o*_ is the theoretical yield of biomass from xylose, *Y*_*P*_ is the theoretical yield of ethanol from xylose and q_*P*_ is the specific productivity of ethanol (g⋅gcel^−1^ h^−1^). The ethanol specific productivity was represented by the Luedeking and Piret equation:

$$\begin{array}{c} q_{P}=\left(\alpha \cdot \mu +\beta \right) \end{array}$$ (8)

Where *α* (g⋅gcel^−1^) and *β* (g⋅gcel^−1^ h^−1^) correspond to the Luedeking and Piret coefficients. It was considered representing in the culture model the *lag* phase of growth and the delay of ethanol production. Thus two delay factors were added to the model as follows [56]:

$$\begin{array}{c} f_{r1}=\frac{q_{1}}{q_{1}+e^{-v_{1}t}};\;\;q_{1}=k\cdot \mu_{max} \end{array}$$ (9)

$$\begin{array}{c} f_{r2}=\frac{q_{2}}{q_{2}+e^{-v_{2}t}} \end{array}$$ (10)

Where, *f*_*r*1_ is the delay factor associated to growth, *q*_1_ is the delay parameter associated to growth, *v*_1_ is the exponent parameter associated to growth, *f*_*r*2_ is the delay factor associated to ethanol production, *q*_2_ is the delay parameter associated to ethanol production and *v*_2_ is the delay parameter associated to ethanol production.

Therefore, Eqs (6) and (8) can be modified to represent production lag:

$$\begin{array}{c} \mu =\frac{S}{S+K_{s}}\cdot f_{r1} \end{array}$$ (11)

$$\begin{array}{c} q_{P}=\left(\alpha \cdot \mu +\beta \right)\cdot f_{r2} \end{array}$$ (12)

The change in oxygen concentration can be represented by the following equation:

$$\begin{array}{c} \frac{dC_{L}}{dt}=k_{L}a\left(C^{*}-C_{L}\right)-q_{O_{2}}\cdot x \end{array}$$ (13)

Where, *C*_*L*_ is the oxygen concentration in the liquid (g⋅L^−1^), C* is the oxygen concentration at saturation in water at 30°C (g⋅L^−1^), *k*_*L*_*a* is the volumetric oxygen transfer coefficient (h^−1^), *q*_*O*_2__ is the specific oxygen transfer rate (g⋅gcel^−1^ h^−1^), *x* is the biomass concentration (g⋅L^−1^). Assuming that under oxygen limiting conditions the magnitude of specific oxygen uptake rate is determined by the oxygen transfer rate since it is assumed that all oxygen transferred from gas to liquid phase is consumed, *q*_*O*_2__ may be calculated as follows:

$$\begin{array}{c} q_{O}{}_{2}=\frac{k_{L}aC^{*}}{x} \end{array}$$ (14)

#### Parameter estimation

The culture model parameters *μ*_*max*_, *K*_*s*_, *Y*_*o*_, *m*, *α*, *β*, *q*_1_, *q*_2_, *v*_1_ and *v*_2_ were determined by least squares fitting of the equations to experimental data. Least squares method was implemented in Matlab2012a.

### Batch cultures

Inoculum propagation was conducted using YPX (*Yeast extract Peptone xylose*) medium composed of 7 g⋅L^−1^ of yeast extract, 14 g⋅L^−1^ of peptone and 14 g⋅L^−1^ of xylose. The ethanol production medium consisted of 6.0 g⋅L^−1^ of yeast extract, 0.20 g⋅L^−1^ of (NH_4_)_2_SO_4_, 5.0 g⋅L^−1^ of KH_2_PO_4_, 0.40 g⋅L^−1^ of MgSO_4⋅_ 7H_2_O, 0.038 g⋅L^−1^ de CaCl_2_ and 15 g⋅L^−1^ of xylose. By adding HCl 2N and NaOH 2N, pH was adjusted to 5.0. All mediums and the fermenter were autoclaved at 121°C for 15 min.

### Microorganism

The yeast *Scheffersomyces stipitis* NRRL Y-7124 (Northern Regional Research Laboratory USDA-ARS, Peoria, Illinois, EEUU) was used for all batch cultures.

### Inoculum preparation

Inoculum propagation was performed from cells previously stored in 1.5 mL cryovials at −80°C YPX medium with 50% V/V glycerol. The propagation was conducted in YPX medium Erlenmeyer flasks with 500 mL culture volume of 150 mL on a rotary shaker incubator (Daihan Labtech Co., LSI-3016R model) with temperature controlled to 30°C and agitation of 250 rpm.

### Batch cultures in fermenter

An Applikon-Biotechnology model *BioBundle Cultivation Systems w/ ez-control* fermenter was used. Agitation was applied using an Rushton turbine impeller, the inlet air flow was controlled by a mass flow controller (MFC), pH was controlled by supplying NaOH 2 N with a peristaltic pumps integrated to the control system and temperature was controlled with a heating jacket. Relative oxygen concentration was measured using a polarographic oxygen sensor. Cultures were carried out at 30°C, 1 L of culture volume and pH 5.

### Sample taking and biomass measurement

Sampling was performed using a sample taking connected to a syringe of 5 mL. The biomass concentration was estimated by turbidimetry measuring absorbance at 620 nm in a spectrophotometer (Jenway, model 6705).

### Sample preparation for HPLC measurement

From each sample 300 *μ*L were centrifuged at 12,000 rpm for 10 min, then supernatant was filtered through a membrane of 0.22 *μ*m pore size and 200 *μ*L were deposited in glass vials.

### Quantification xylose and ethanol content by HPLC

Xylose and ethanol concentrations were measured by high-performance liquid chromatography (HPLC). An HPLC system Agilent Model 1260 *Infinity Quaternary LC System* was used with UV/IR detector and automatic sample injection. A column Aminex BioRad HPX-97H and H_2_SO 5 mM as mobile phase were used, conditions were 0.6 mL⋅min^−1^ as flow and temperature of 45°C. Retention times were 9.5 and 21.3 min for xylose and ethanol, respectively.

### Determination of the volumetric oxygen transfer coefficient (*k*_*L*_*a*)

The absorption method described by García-Ochoa *et al*. [57] which consists of removing oxygen from the liquid phase and then begin aeration measuring the concentration of dissolved oxygen was used. To remove oxygen sodium sulfite and copper sulfate were added. Two conditions of limited oxygen supply were considered with inlet air flow of 3 L⋅min^−1^; a condition with *k*_*L*_*a* = 25 h^−1^ established at 250 rpm and another with *k*_*L*_*a* = 15 h^−1^ established at 150 rpm.

## Results

### Phenotypic phase plane analysis

Phenotypic Phase Plane (PhPPs) analysis [44] was used to obtain the specific growth rate (*μ*) as a function of the oxygen (q_*O*_2__) and xylose (q_*xyl*_) uptake rates. A single phenotypic phase is defined as the region where the derivative of *μ* with respect to q_*xyl*_ and q_*o*_2__ is constant. Each single phenotypic phase is associated to a particular active set of reactions (non-zero fluxes). On the other hand, each single point on a given phenotypic phase corresponds to a metabolic state, which is characterized by the flux values of the active set.

**Fig 1:**
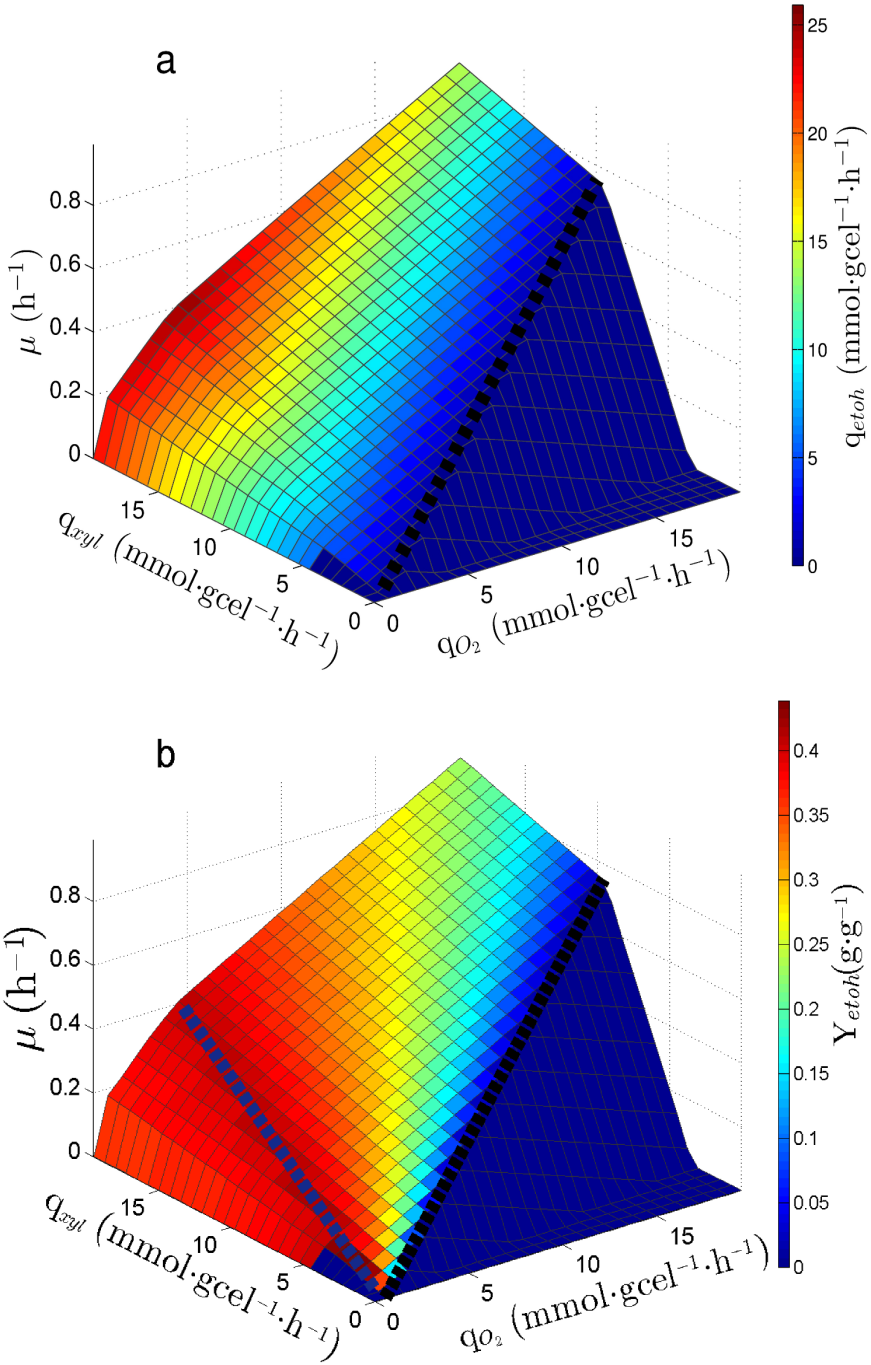
Phenotypic Phase Plane (PhPP) for *S. stipitis*. (a): PhPP shows *q*_*etoh*_ as colored contours. (b): PhPP shows *Y*_*etoh*_ as colored contours. Dashed black line: line of optimality for biomass yield. Blue dashed line: line of optimality for ethanol yield.

As shown in Fig 1a and 1b, along with *μ* the specific ethanol productivity (q_*etoh*_) and the ethanol yield from xylose (Y_*etoh*/*xyl*_) are shown over the PhPP as colored contours, respectively. Three relevant subsets of metabolic states can be distinguished on the PhPP (see Fig 1a): *i*) The line of optimality for biomass production, which is represented by the dashed black line, where the uptake rates for oxygen and xylose are in exact proportion to allow maximum biomass yield. *ii*) The phenotypic phase to the right hand side of the dashed black line, where growth is limited by xylose uptake rate. *iii*) The phenotypic phases to the left hand side of the dashed black line, where oxygen uptake rate is below the value necessary to achieve fully aerobic growth and ethanol is produced. The dashed blue line between the contours of Y_*etoh*/*xyl*_ in Fig 1b, corresponds to the line of optimality for the ethanol yield. Metabolic states associated to subsets *i* and *ii* do not allow ethanol production, corresponding to fully aerobic metabolism. On the other hand, subset *iii* corresponds to fermentative metabolism since ethanol is produced.

### Sensitibity of ethanol production to the Available Reducing Capacity (ARC) in *S. stipitis*

The sensitivity of q_*etoh*_ to changes on ARC is referred as $\lambda_{ARC}^{etoh}$ (see methods section for details). One of the following three cases can be obtained from the analysis: *i*) Increasing ARC generates an increment in q_*etoh*_, then ARC limits ethanol production. *ii*) q_*etoh*_ decreases when increasing ARC, thus ARC is in excess for ethanol production. *iii*) Changes in ARC do not affect q_*etoh*_. Whether case *i*, *ii* or *iii* occurs is determined as follows (see methods section for details): If $\lambda_{ARC}^{etoh}$ is less than zero ARC limits ethanol production, if $\lambda_{ARC}^{etoh}$ is greater than zero ARC is in excess for ethanol production and if $\lambda_{ARC}^{etoh}$ is equal to zero, changes in ARC do not improve ethanol production.

**Fig 2:**
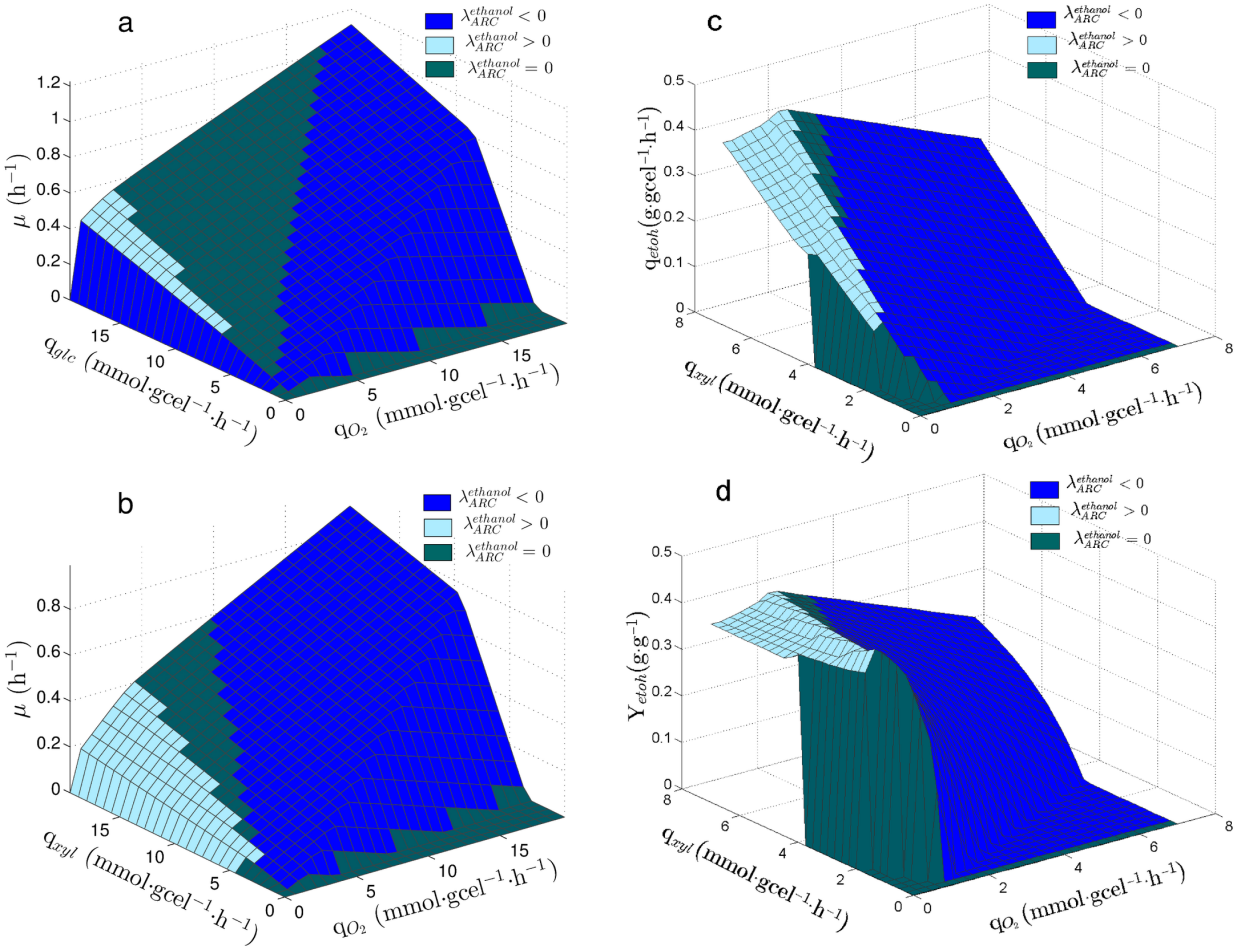
Sensitivity of ethanol production on the PhPP of *S. stipitis*. $\lambda_{ARC}^{etoh}$: Sensitivity of q_*etoh*_ to the Available Reducing Capacity (ARC). Planes are colored according to $\lambda_{ARC}^{etoh}$ value, indicating if ARC is limiting ($\lambda_{ARC}^{etoh}<0$), is in excess ($\lambda_{ARC}^{etoh}>0$) or it has no effect on q_*etoh*_. (a) PhPP for the growth rate (*μ*) using glucose. (b) PhPP for the growth rate (*μ*) using xylose. (c) PhPP for the specific productivity of ethanol (q_*etoh*_) using xylose. (d) PhPP for the yield of ethanol from xylose (Y_*etoh*_).

Fig 2a and 2b show $\lambda_{ARC}^{etoh}$ values over the PhPP of *S. stipitis* using⋅glucose and xylose, respectively. The following regions can be observed starting from the line of optimality for biomass toward the left: First, a region where ARC limits ethanol production ($\lambda_{ARC}^{etoh}<0$), then, a region where ARC does not affect ethanol production($\lambda_{ARC}^{etoh}=0$) and finally, a region showing excess in ARC for ethanol production ($\lambda_{ARC}^{etoh}>0$). The PhPP using xylose shown in Fig 2b presents the same regions. It can be observed that the region having $\lambda_{ARC}^{etoh}<0$ is wider when using xylose (Fig 2b) than when using⋅glucose (Fig 2a) as carbon source, so that the number of metabolic states where ethanol production is limited by ARC increase when xylose is used. It can also be seen that the region showing $\lambda_{ARC}^{etoh}>0$ is greater in Fig 2b than in Fig 2a, meaning that the number of metabolic states having excess in ARC also increase when using xylose. The expansion of both regions, $\lambda_{ARC}^{etoh}<0$ and $\lambda_{ARC}^{etoh}>0$, narrows the zone which shows $\lambda_{ARC}^{etoh}=0$, thus the number of metabolic states where ARC does not affect ethanol production decrease when using xylose.

On the other hand, Fig 2c and 2d show $\lambda_{ARC}^{etoh}$ values over the PhPP for the ethanol production (q_*etoh*_) and the PhPP for the ethanol yield from xylose of *S. stipitis* using⋅glucose and xylose, respectively. It can be seen that the line of optimality for ethanol production is over the line that divides the regions where $\lambda_{ARC}^{etoh}<0$ and $\lambda_{ARC}^{etoh}=0$ (Fig 2c and 2d).

#### Sensitibity of ethanol production to ARC in *S. cerevisiae*

**Fig 3:**
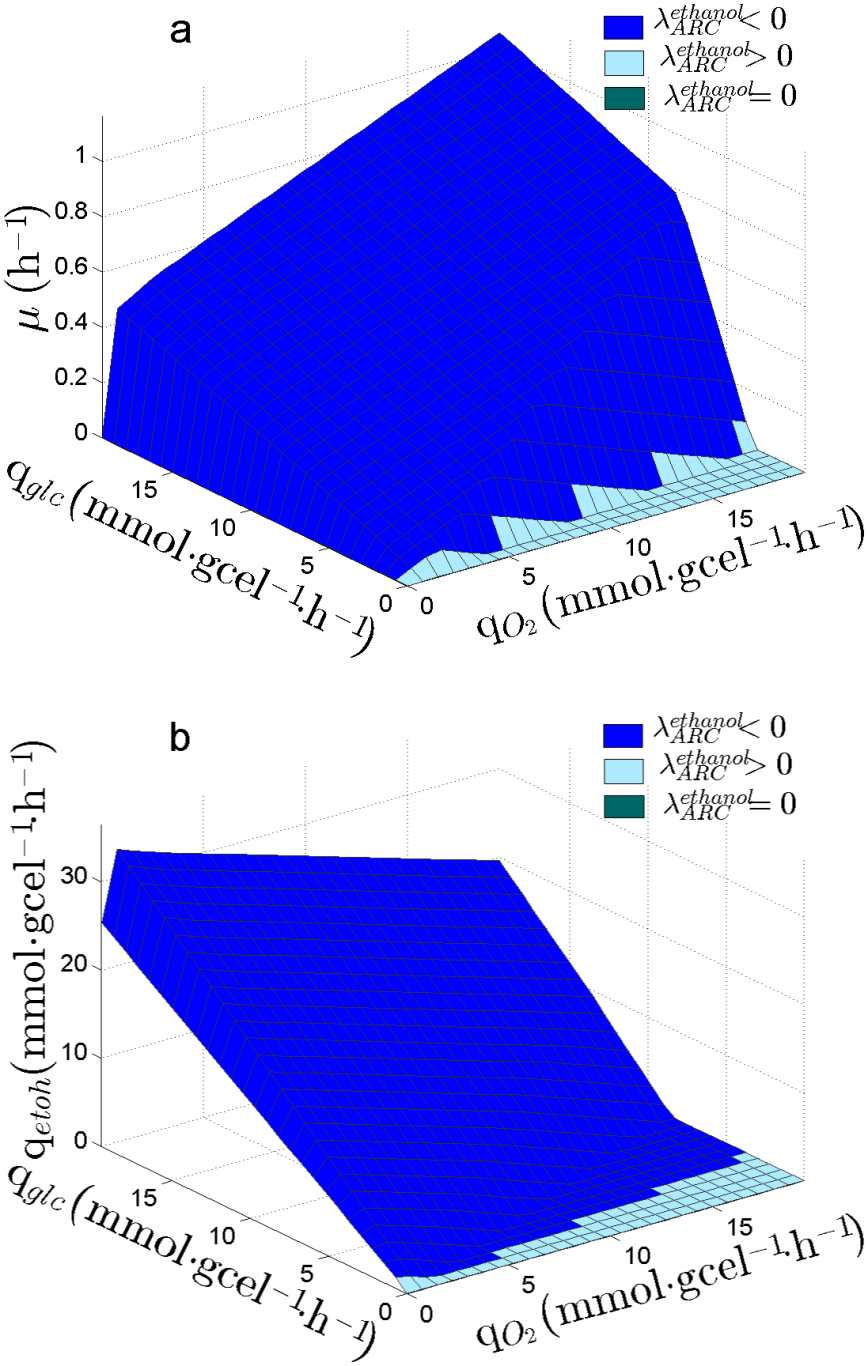
Sensitivity of ethanol production on the PhPP of *S. cerevisiae*. $\lambda_{ARC}^{etoh}$: Sensitivity of q_*etoh*_ to the Available Reducing Capacity (ARC). Planes are colored according to $\lambda_{ARC}^{etoh}$ value, indicating if ARC is limiting ($\lambda_{ARC}^{etoh}<0$), is in excess ($\lambda_{ARC}^{etoh}>0$) or it has no effect on q_*etoh*_. (a) showing the PhPP for specific growth rate (*μ*). (b) showing PhPP for specific ethanol productivity (*q*_*etoh*_).

The PhPP of *S. cerevisiae* was also determined in order to compare it with the PhPP of *S. stipitis*. As Fig 3a and 3b show, for *S. cerevisiae* $\lambda_{ARC}^{etoh}$ is less than zero through the entire PhPP, then an increment in ARC always leads to an increase in ethanol production.

The sensitivity analysis of the both yeasts indicates that in *S. stipitis*, unlike *S. cerevisiae*, an increment in ARC does not always increase ethanol production,

### PhPP and sensitivity analysis with metabolic improvements for ethanol production

**Fig 4:**
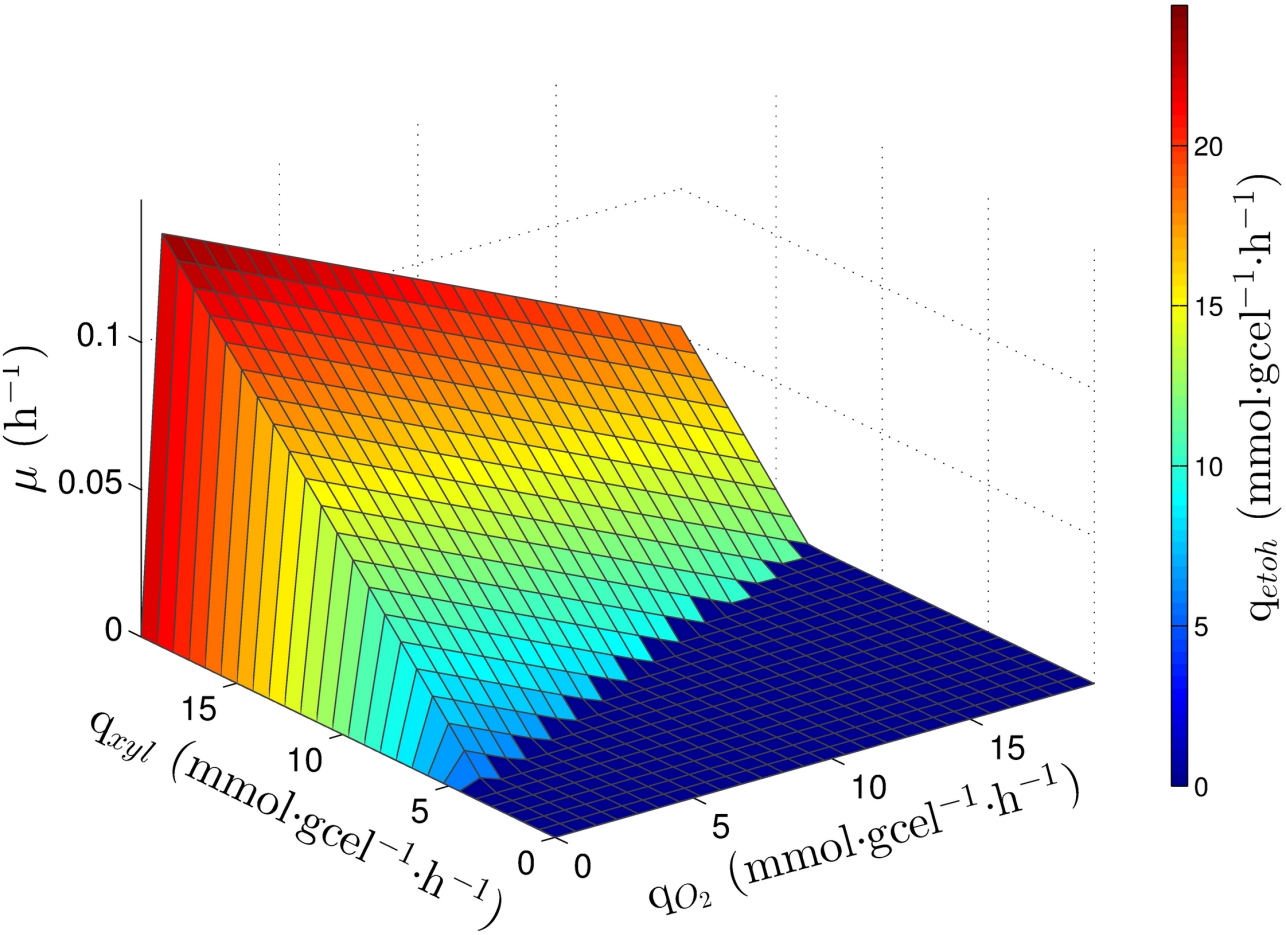
PhPP of *S. stipitis* having eliminated reactions. PhPP shows the response of *μ* and q_*etoh*_ having eliminated AOX and COX.

Previous work by Acevedo *et al*. [46], reported *in-silico* reaction deletions able to associate ethanol production (q_*etoh*_) to biomass production (*μ*) in *S. stipitis*, all of them related to the respiratory chain. Fig 4 shows the PhPP of *S. stipitis* with the cytochrome oxidase (COX) and the alternative oxidase (AOX) deleted. It can be seen that the highest values for *μ* and q_*etoh*_ coincide, the same was observed for the other deletions.

**Fig 5:**
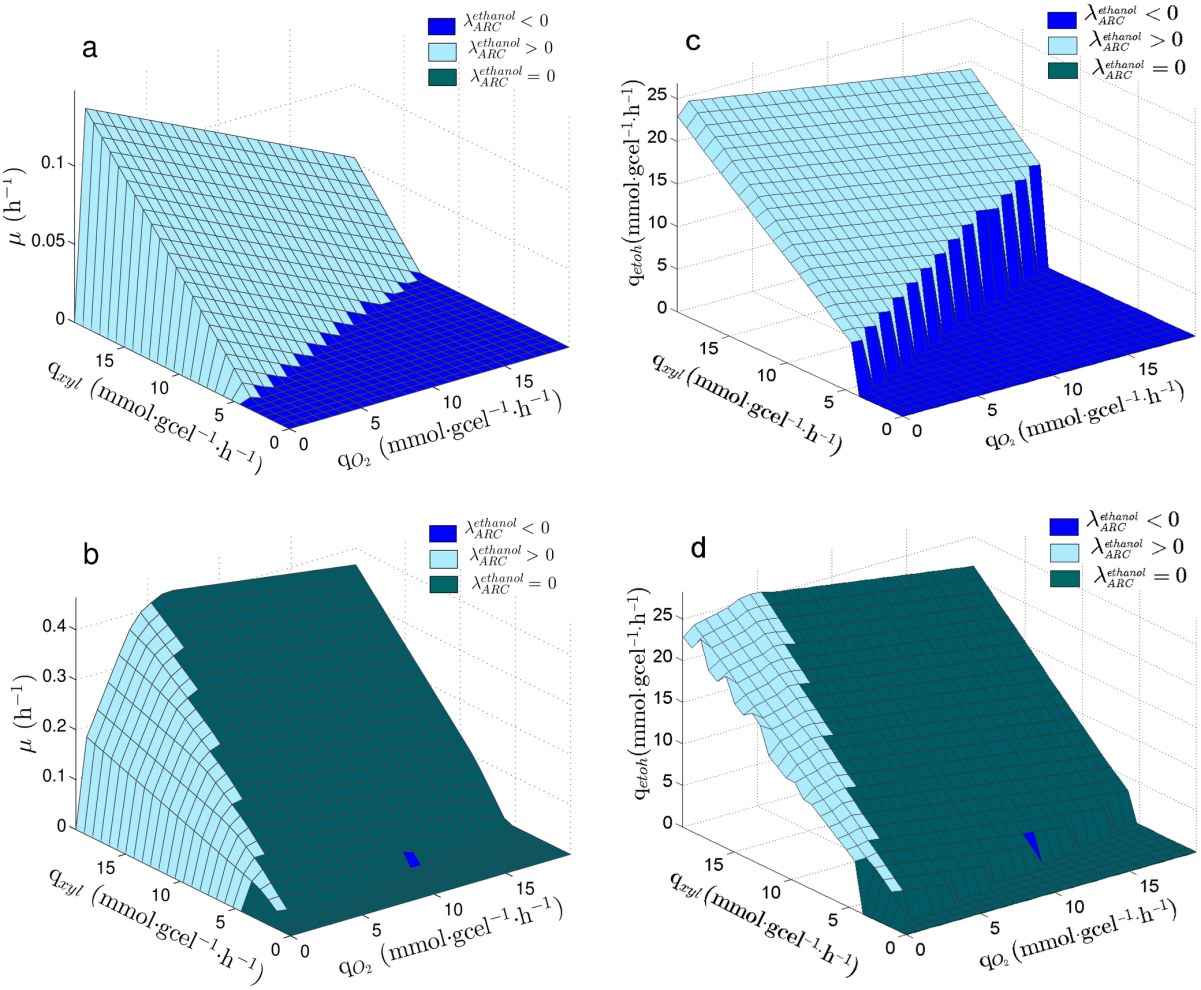
PhPP of *S. stipitis* showing $\lambda_{ARC}^{etanol}$. (a): Reactions eliminated. (b): Reactions inhibited by 80%. (c) Shows PhPP for specific ethanol production (*q*_*etoh*_) for reactions eliminated. (d) Shows PhPP for specific ethanol production (*q*_*etoh*_) for 80% inhibition.

On the other hand, sensitivity analysis showed that there is an excess of ARC for ethanol production through all the PhPP (Fig 5a and 5c). Hence, eliminating respiratory components decreases in such a way the NADH oxidative capacity that ethanol production is negatively affected by the excess of ARC. Thereby, despite achieving coupling between ethanol and biomass production, the metabolic response obtained was unfavorable for ethanol production. Since the latter results showed a severe effect on ethanol production, a sensitivity analysis to characterize the effect of the partial inhibition of both COX and AOX on ethanol production was performed. As seen in Fig 5b and 5d, the sensitivity equals to zero through the entire PhPP when COX and AOX are inhibited, then ARC is not having a negative effect on ethanol production. Therefore, inhibiting the respiratory chain is a promisory strategy to improve ethanol production from xylose.

### Inhibition of the respiratory chain in batch cultures of *S. stipitis*

Experimental essays to determine whether the inhibition of the respiratory chain improves ethanol production in *S. stipitis* were done. Inhibitors of the respiratory chain were added to batch cultures of *S. stipitis* using xylose at conditions of limited oxygen supply. Salicylhydroxamic acid (SHAM) was used to inhibit AOX, while potassium cyanide and sodium azide were used to inhibit COX. As shown in Table 1, inhibitors decreased the yield. In the case of SHAM, the yield decreased more than 25%, while cyanide and sodium azide decreased yield over 50%. Considering that all previously determined *in-silico* deletions corresponded to the respiratory chain, the inhibition of the NADH dehydrogenase complex I was also considered. As it can be seen in Table 1, the addition of rotenone for inhibiting Complex I increased yield up to 13%.

**Table 1:** Inhibitory essays in flasks.

Enzyme	Inhibition	Y_*etoh*/*xyl*_ (g⋅g^−1^)	Q_*etoh*_ (g⋅L^−1^⋅h^−1^)
Control	Control	0,3006 ± 0,0090	0,1576 ± 0,0058
COX	Potassium cyanide (1 mM)	0,1068 ± 0,0270	0,0359 ± 0,0072
COX	Sodium azide(0,01 mM)	0,0948 ± 0,0347	0,0330 ± 0,0125
OXA	SHAM (1 mM)	0,2240 ± 0,0357	0,1059 ± 0,0158
COX + OXA	SHAM (1 mM) + Potassium cyanide (1 mM)	0,1173 ± 0,0328	0,0375 ± 0,0098
COX + OXA	SHAM (1 mM) + Sodium azide (0,01 mM)	0,1186 ± 0,0236	0,0397 ± 0,0085
CINADH	Rotenone (0,25 mM)	0,3453 ± 0,0205	0,1169 ± 0,0031

COX: cytochrome oxidase. OXA: alternative oxidase.

CINADH: NADH dehydrogenase complex I. SHAM: Salicylhydroxamic acid.

Y_*etoh*/*xyl*_: ethanol yield from xylose. Q_*etoh*_: ethanol volumetric productivity.

### Essays in fermenter inhibiting the NADH dehydrogenase Complex I

**Fig 6:**
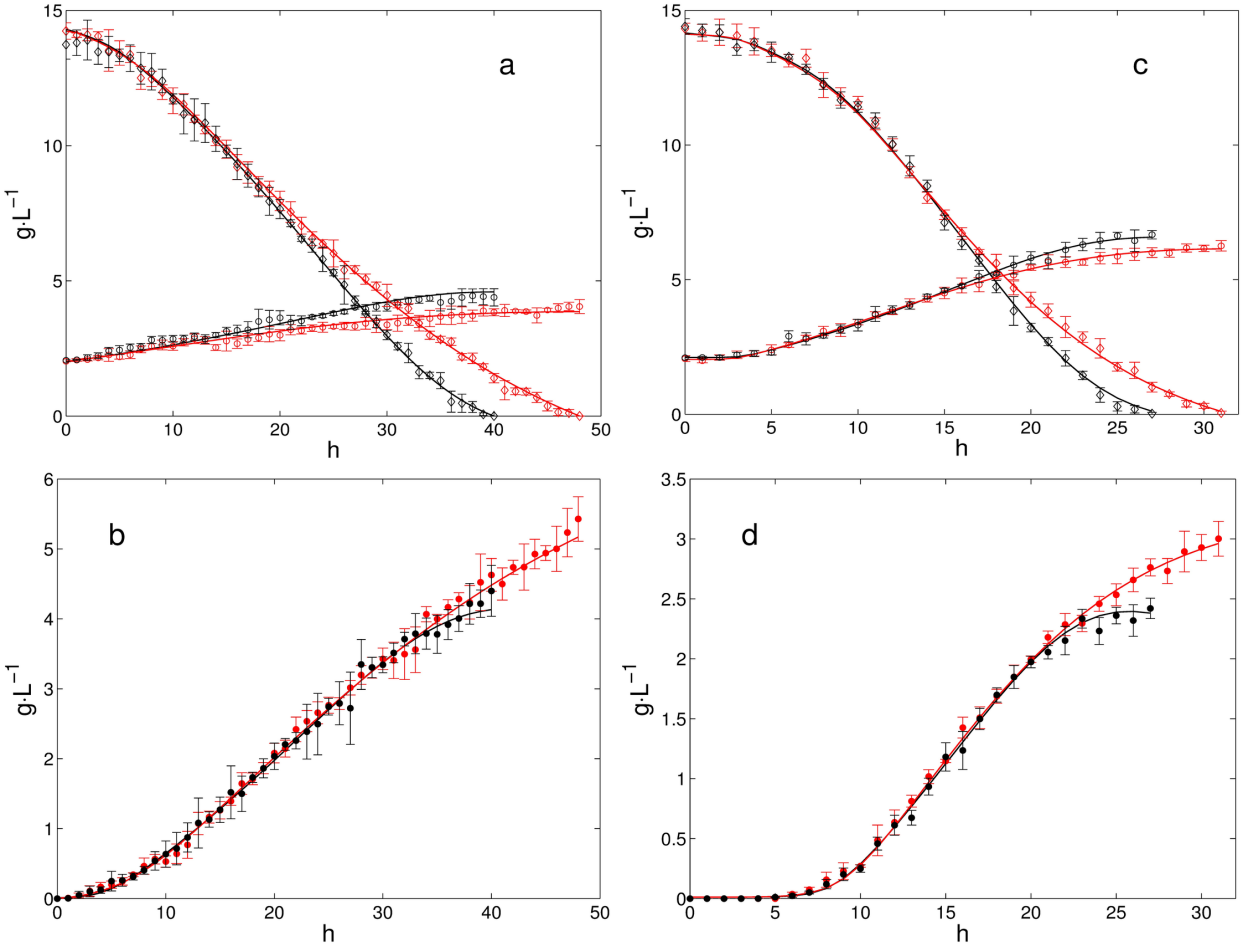
Inhibitory assays in batch cultures. Black: Cultures without rotenone. Red: Cultures with rotenone. Open circles: Biomass concentration. Filled circles: Ethanol concentration. Open diamonds: Xylose concentration. (a): Xylose consumption and biomass production at *k*_*L*_*a* = 15 h^−1^. (b): Ethanol production at *k*_*L*_*a* = 15 h^−1^. (c): Xylose consumption and biomass production at *k*_*L*_*a* = 25 h^−1^. (d): Ethanol production at *k*_*L*_*a* = 25 h^−1^. Solid lines: Culture model. All experiences were repeated five times, error bars are shown.

Batch cultures in fermenter with limited oxygen supply considering *k*_*L*_*a* = 25 h^−1^ and *k*_*L*_*a* = 15 h^−1^ were performed. Fig 6 shows the concentrations of xylose, biomass and ethanol over time. Data in red color corresponds to cultures with rotenone. Cultures at *k*_*L*_*a* = 15 h^−1^ are shown in Fig 6a and 6b, while results at *k*_*L*_*a* = 25 h^−1^ are shown in Fig 6c and 6d. It can be seen that rotenone addition extends the time in which xylose is completely consumed (Fig 6a and 6c). Moreover, as Fig 6b and 6d show, final ethanol concentration increases when inhibiting. Yield (Y_*etoh*/*xyl*_) increases by ∼24% at *k*_*L*_*a* = 25 h^−1^ and by ∼18% at *k*_*L*_*a* = 15 h^−1^ (Table 2). On the other hand, productivity (Q_*etoh*_) decreased ∼41% and ∼35% at *k*_*L*_*a* = 25 h^−1^ and *k*_*L*_*a* = 15 h^−1^, respectively.

**Table 2:** Inhibitory essays in fermenter using rotenone.

culture	k_*L*_a (h^−1^)	Y_*etoh*/*xyl*_ (g⋅g^−1^)	Q_*etoh*_ (g⋅L^−1^⋅h^−1^)
Control	25	0,1681 ± 0,0044	0,3026 ± 0,0108
Rotenone (2,5 mM)	25	0,2096 ± 0,0096*	0,1766 ± 0,0085*
Control	15	0,3207 ± 0,0300	0,4888 ± 0,0405
Rotenone (2,5 mM)	15	0,3812 ± 0,0215*	0,3193 ± 0,0187*

Y_*etoh*/*xyl*_: Ethanol yield from xylose.

Q_*etoh*_: Ethanol volumetric productivity.

k_*L*_a: Volumetric oxygen transfer coefficient.

*: Significant difference from control (*p* < 0,05)

### Determination of uptake rates from the batch cultures

**Fig 7:**
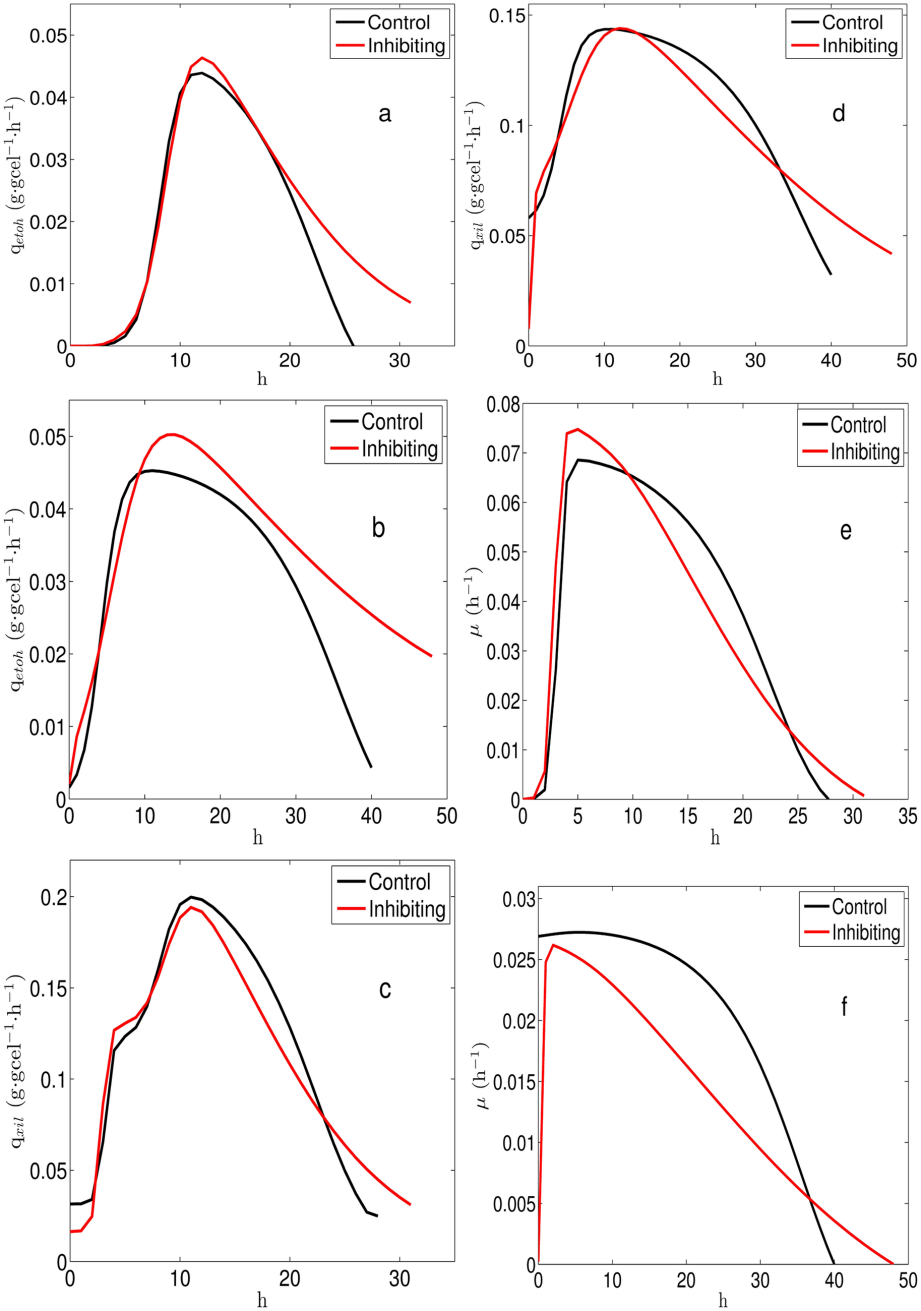
Trajectories obtained from the culture model. (a): Ethanol specific productivity at *k*_*L*_*a* = 25 h^−1^. (b): Ethanol specific productivity at *k*_*L*_*a* = 15 h^−1^. (c): Xylose specific uptake rate at *k*_*L*_*a* = 25 h^−1^. (d): Xylose specific uptake rate at *k*_*L*_*a* = 15 h^−1^. (e): Specific growth rate at *k*_*L*_*a* = 25 h^−1^. (f): Specific growth rate at *k*_*L*_*a* = 15 h^−1^.

**Fig 8:**
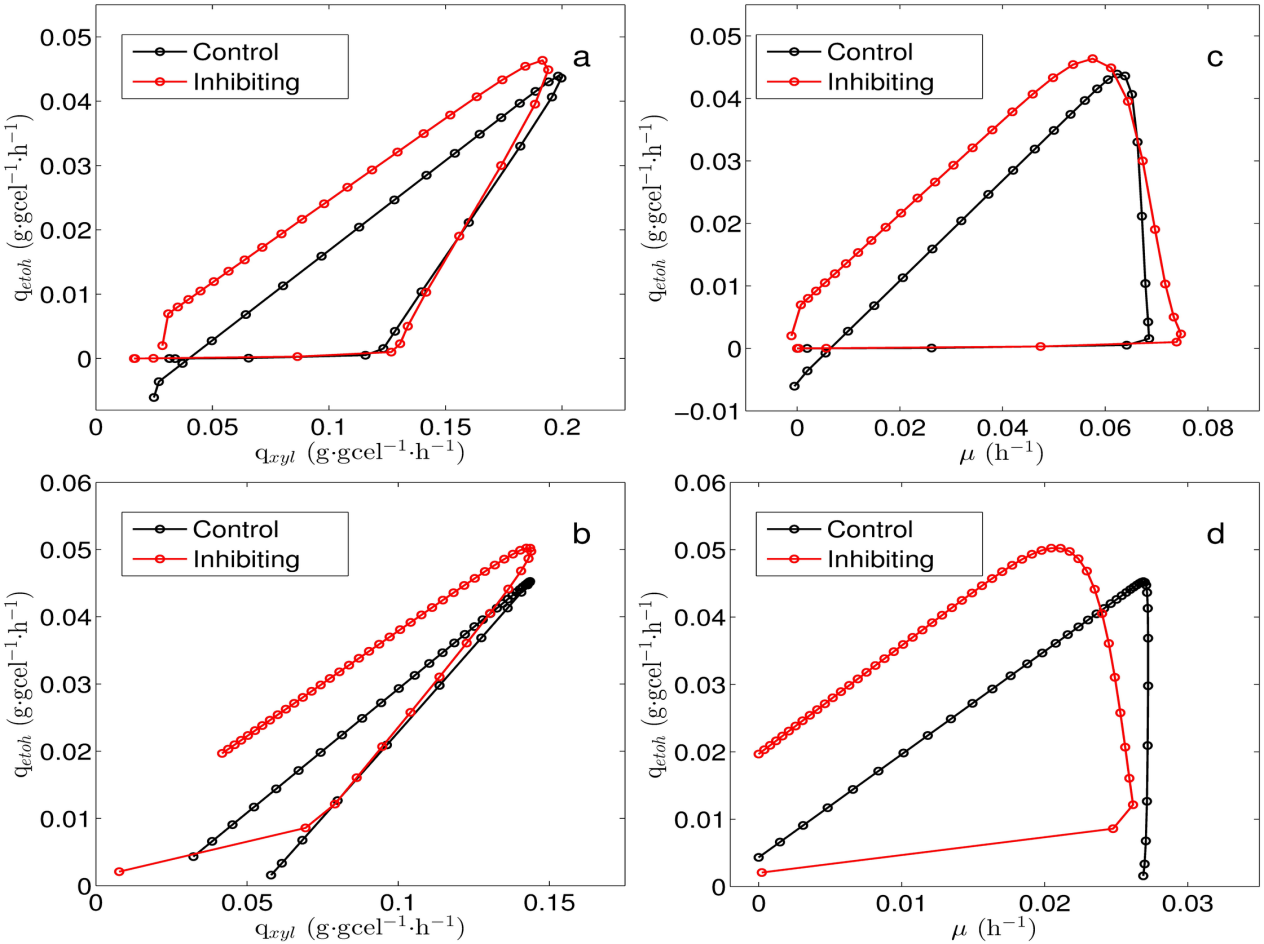
Ethanol production trajectory with respect to xylose uptake rates and growth. (a): Ethanol specific productivity respect to xylose specific uptake rate at *k*_*L*_*a* = 25 h^−1^. (b): Ethanol specific productivity respect to xylose specific uptake rate at *k*_*L*_*a* = 15 h^−1^. (c): Ethanol specific productivity respect to specific growth rate at *k*_*L*_*a* = 25 h^−1^. (d): Ethanol specific productivity respect to specific growth rate at *k*_*L*_*a* = 15 h^−1^.

Xylose and oxygen uptake rates which were incorporated to the FBA to obtain the metabolic states associated to the cultures were calculated from the experimental data. To estimate xylose uptake rate a kinetic model describing xylose, biomass and ethanol concentration was used. Specific ethanol productivity and specific growth rate were also obtained from the kinetic model. On the other hand, to estimate oxygen uptake rate a balance equation for dissolved oxygen was considered and conditions of limited oxygen supply were assumed (see methods section for details). Solid lines in Fig 6 correspond to xylose, biomass and ethanol concentrations resulting from the fitting of the kinetic model to the experimental data. Xylose specific uptake rate, ethanol specific productivity and biomass specific growth rate obtained from the culture model are shown in Fig 7. As it can be observed in Fig 7a and 7b, values for q_*etoh*_ are slightly higher in cultures with rotenone (red lines), this effect is more pronounced at k_*L*_a = 15h^−1^ (Fig 7b). Fig 7c and 7d show q_*xyl*_, here, it can be noted that rotenone decreases q_*xyl*_. Similarly, Fig 7e and 7f show that *μ* decreases when adding rotenone. Besides, Fig 8 shows q_*etoh*_ relative to q_*xyl*_ and *μ*, it can be seen that rotenone increases ethanol production for all cases (Fig 8a–8d).

### Characterization of phenotypic phases associated to culture trajectories

**Fig 9:**
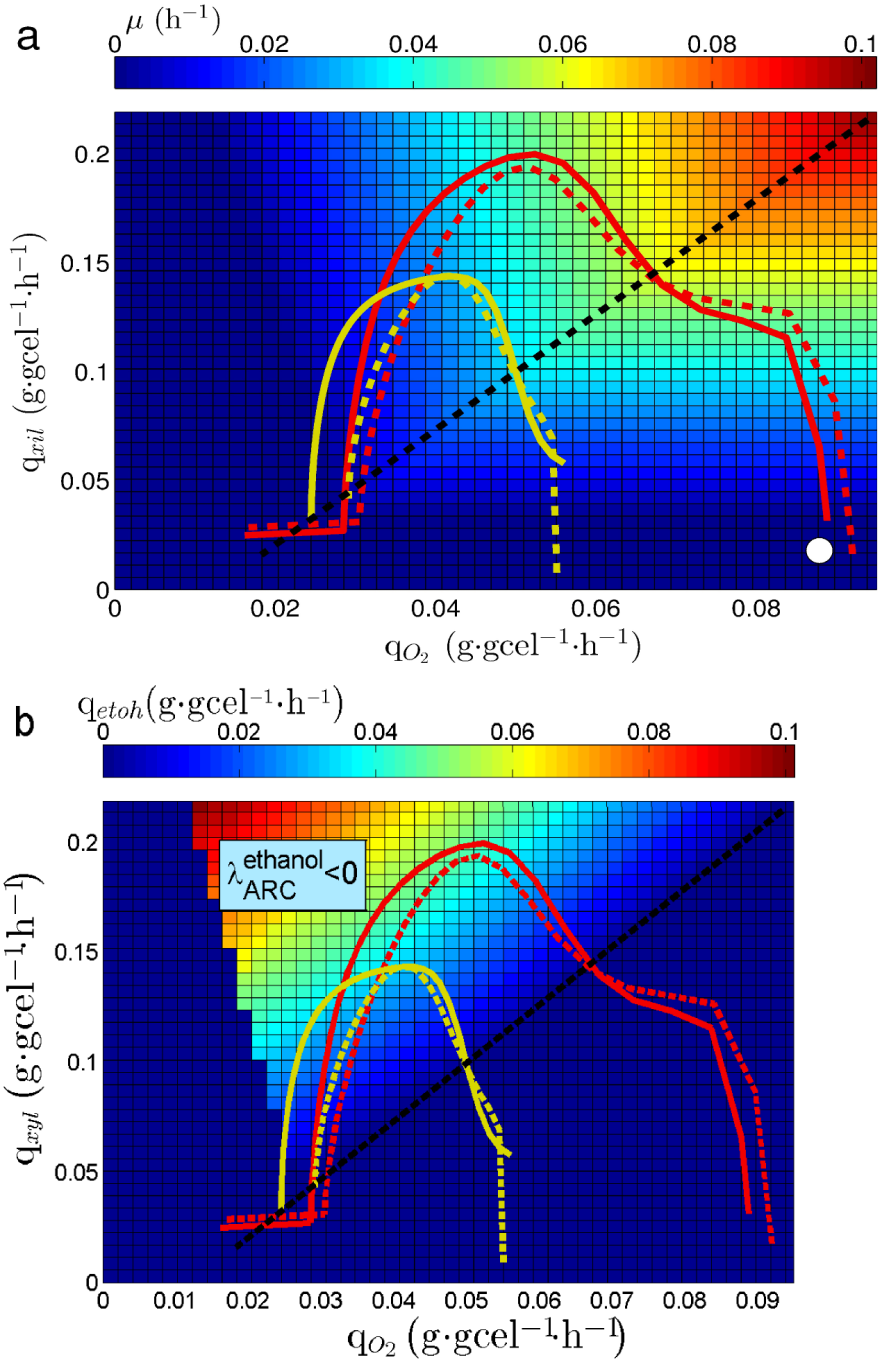
Culture trajectories on the PhPP. Solid yellow line: trayectory at k_*L*_a = 15 h^−1^ with no inhibition. Dashed yellow line: trayectory at k_*L*_a = 15 h^−1^ with inhibition. Solid red line: trayectory at k_*L*_a = 25 h^−1^ with no inhibition. Dashed red line: trayectory at k_*L*_a = 25 h^−1^ with inhibition. Dashed black line: line of optimality for biomass yield. (a): Colored contours for *μ*. (b): Colored contours for q_*etoh*_. White circle indicates the start of trajectories.

With the purpose of characterizing the metabolic states associated to the batch cultures, the values of q_*xyl*_ and q_*O*_2__ were plotted over the PhPP, this allowed to identify trajectories on the phenotypic phases. As neither xylitol nor acetic acid was detected under the studied conditions, the possible effect of the xylitol production on the balance of redox cofactors can be left aside. Then the effect of the respiratory chain, in this case the Complex I NADH Dehydrogenase, can be considered as the main contributor to changes in redox balance. Fig 9a and 9b show resulting trajectories over the colored contours for *μ* and q_*etoh*_, respectively. All trajectories cover the three subsets previously defined over the PhPP, starting from subset *ii* (white circle in Fig 9a illustrates a starting point), then crossing subset *i*, transiting over subset *iii* and finally ending close to subset *i*. It can be observed in Fig 9a that trajectories at k_*L*_*a* = 15 h^−1^ (solid and dashed yellow lines) are associated to lower values for *μ* than the trajectories at k_*L*_*a* = 25 h^−1^ (solid and dashed red lines). According to the colored contours of the PhPP in Fig 9a, trajectories at k_*L*_*a* = 15 h^−1^ reaches a specific growth rate of about 0.035 h^−1^, while trajectories at k_*L*_*a* = 25 h^−1^ reaches about 0.06 h^−1^. These values predicted by the PhPP are close to the values for *μ* obtained from the batch cultures at k_*L*_*a* = 25 h^−1^ and at k_*L*_*a* = 15 h^−1^ shown in Fig 7e and 7f, respectively. Besides, as seen in Fig 9b, trajectories at k_*L*_*a* = 15 h^−1^ reaches an ethanol specific productivity about 0.040 mmol⋅gcel^−1^⋅h^−1^ of, while at k_*L*_*a* = 25 h^−1^ about 0.050 is obtained over the PhPP. These values from the PhPP are close to the values for q_*etoh*_ calculated from the cultures at k_*L*_*a* = 25 h^−1^ and at k_*L*_*a* = 15 h^−1^ shown in Fig 7a and 7b, respectively. Regarding the effect of the rotenone it can be noted that trajectories with inhibition (yellow and red dashed lines in Fig 9) tend to be closer to the optimality line for biomass. Nonetheless, considering the dispersion of the experimental data, these differences are minor when compared to the controls. Also, the $\lambda_{ARC}^{etoh}$ associated to subset *iii* is shown in Fig 9b. Here, as $\lambda_{ARC}^{etoh}$ is less than zero, an increase in q_*etoh*_ is predicted if ARC increases. Therefore, considering that inhibiting complex I NADH dehydrogenase implies an increase in ARC, the latter sensitivity predicts that ethanol production should improve.

### Determination of $\lambda_{ARC}^{etoh}$ and $\lambda_{ARC}^{biomass}$ in trajectories with and without inhibition

**Fig 10:**
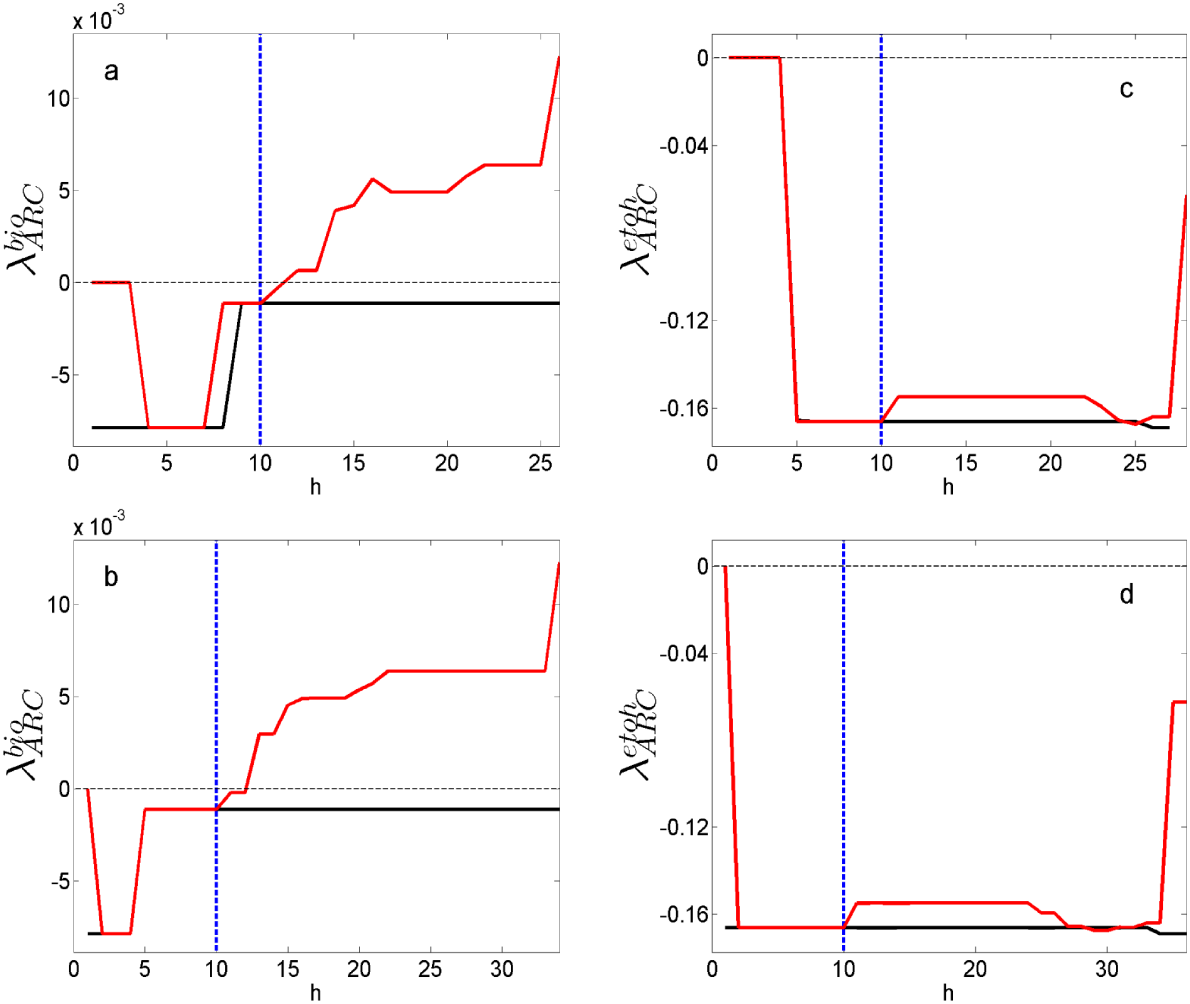
Trajectories of sensitivities. Solid red line: trajectory for $\lambda_{ARC}^{bio}$ and $\lambda_{ARC}^{etoh}$ with inhibition. Solid black line: trayectories without inhibition. Dashed blue line: inhibition starting time. Dashed black line: zero coordinate in *Y* axis. (a) $\lambda_{ARC}^{bio}$ for k_*L*_a = 25 h^−1^. (b) $\lambda_{ARC}^{bio}$ for k_*L*_a = 15 h^−1^. (c) $\lambda_{ARC}^{etoh}$ for k_*L*_a = 25 h^−1^. (d) $\lambda_{ARC}^{etoh}$ for k_*L*_a = 15 h^−1^.

The results shown in Fig 9b predicts an increase in ethanol production as ARC increases, however, the value of $\lambda_{ARC}^{etoh}$ only predicts a response of q_*etoh*_ but it does not characterize subsequent metabolic states with an inhibited complex I. Then it is necessary to analyze the subsequent metabolic states considering the inhibition of complex I, in order to verify whether changes in sensitivities are consistent with states favoring ethanol production. To this purpose, q_*xyl*_ and q_*O*_2__ trajectories were used as constraints for the FBA and the inhibition were simulated by applying a constraint on the upper bound of the flux associated to complex I. Thus by using FBA metabolic states were successively calculated and the sensitivities associated to the trajectories were obtained. Along with $\lambda_{ARC}^{etoh}$, the sensitivity for biomass production to ARC ($\lambda_{ARC}^{biomass}$) was also obtained, allowing a more complete analysis of the metabolic states with inhibition. Fig 10a and 10b show the trajectory for $\lambda_{ARC}^{biomass}$ with (red line) and without (black line) inhibition. In both cases, it can be observed that after inhibiting the values for $\lambda_{ARC}^{biomass}$ increases up to a point of going from negative to positive, hence, ARC changes from being limiting to be in excess for biomass production. On the other hand, $\lambda_{ARC}^{etoh}$ remains negative after de inhibition (Fig 10c and 10d), meaning that increasing ARC improves ethanol production. These results suggest that inhibiting complex I increments ARC to the point of negatively affecting biomass production and favoring ethanol production.

### Flux change comparison between inhibited and non-inhibited metabolism

Changes in the flux-carrying set (FCS) were analyzed in order to explain a change in the ethanol production generated by the inhibition of the CINADH. The FCS of a metabolic state with inhibited the CINADH was compare against the FCS of a metabolic state without inhibition in order to obtain the changes in the FCS caused by the inhibition of the CINADH.

According to the analysis made, when CINADH is inhibited, NADH becomes available to other reactions, including ethanol production reaction. This does not mean that ethanol flux would increase significantly because its production is dependent as well on carbon flux. Table 3 shows the fluxes that decreased the most, it can be seen that these reactions are related to the energy metabolism. Tables 4 and 5 shows the fluxes that increased the most, as seen, a great extent of them correspond to glycolysis and the Krebs cycle. These results show a redistribution of those fluxes, favouring carbon flux towards ethanol flux. This is in accordance with a previous report by Jeffries et al. 2007 [7] for S. stipitis grown on xylose where changes in the expression of the main enzymes of the central carbon metabolism were analized under fully aerobic and oxygen-limited (ethanol-producing). It was found that the increased in ethanol production was associated with the up-regulation of several enzymes of the glycolysis, which coincides with what it is show on Tables 4 and 5.

**Table 3:** Reaction showing the largest flux reduction after CINADH inhibition.

Absolute flux shift mmol gcel^−1^ h^−1^	Reaction name	Reaction formula	Related metabolism
13,9370	NADH dehydrogenase with H+ transport	h[m] + q6[m] + nadh[m] + 4 h+[m] → nad[m] + q6h2[m] + 4 h+[c]	Oxidative Phosphorylation
0,6695	ATP synthase mitochondrial	adp[m] + pi[m] + 4 h+[c] → h2o[m] + atp[m] + 4 h+[m]	Oxidative Phosphorylation
0,6506	ADP/ATP transport mitochondrial	h2o[m] + adp[c] + pi[c] + atp[m] + h+[c] → h2o[c] + atp[c] + adp[m] + pi[m] + h+[m]	Transport Mitochondrial
0,3343	ATPase, cytosolic	h2o[c] + h[c] + atp[c] → h[e] + adp[c] + pi[c]	Oxidative Phosphorylation
0,0532	Inorganic diphosphatase	h2o[c] + ppi[c] → h[c] + 2 pi[c]	Oxidative Phosphorylation
0,0456	adenylate kinase	atp[c] + amp[c] ↔ 2 adp[c]	Purine Metabolism
0,0444	Acetyl-coA Synthetase	atp[c] + coa[c] + ac[c] → amp[c] + ppi[c] + accoa[c]	Glycolysis/ Gluconeogenesis
0,0196	Ammonia reversible transport	nh4[e] ↔ nh4[c]	Transport Reaction
0,0147	Citrate transport Mitochondrial	icit[m] + cit[c] ↔ cit[m] + icit[c]	Transport Mitochondrial

**Table 4:** Reaction showing the largest flux increase after CINADH inhibition.

Absolute flux shift mmol gcel^−1^ h^−1^	Reaction name	Reaction formula	Related metabolism
0,5841	Succinate Dehydrogenase (Ubiquinone) Mitochondrial	q6[m] + succ[m] ↔ q6h2[m] + fum[m]	Citrate cycle (TCA cycle)
0,5315	glycerol-3-phosphate dehydrogenase (NAD)	h[c] + nadh[c] + dhap[c] → nad[c] + glyc3p[c]	Glycerolipid Metabolism
0,3562	isocitrate dehydrogenase (NADP+) Mitochondrial	icit[m] + nadp[m] → co2[m] + akg[m] + nadph[m]	Citrate cycle (TCA cycle)
0,0857	Pyruvate dehydrogenase mitochondrial	coa[m] + pyr[m] + nad[m] → co2[m] + accoa[m] + nadh[m]	Glycolysis/ Gluconeogenesis
0,0837	Valine reversible mitochondrial transport via proton symport	h[c] + val-L[c] ↔ h[m] + val-L[m]	Transport Mitochondrial
0,0837	Valine transaminase mitochondrial	akg[m] + val-L[m] ↔ 3mob[m] + glu-L[m]	Valine, leucine and isoleucine Metabolism
0,083	Valine transaminase	akg[c] + val-L[c] ↔ 3mob[c] + glu-L[c]	Valine, leucine and isoleucine Metabolism
0,0577	Succinate-CoA ligase (ATP-forming), mitochondrial	atp[m] + coa[m] + succ[m] ↔ adp[m] + pi[m] + succoa[m]	Citrate cycle (TCA cycle)
0,0577	2-Oxoglutarate Dehydrogenase (Mitochondrial)	coa[m] + akg[m] + nad[m] → co2[m] + succoa[m] + nadh[m]	Citrate cycle (TCA cycle)
0,0494	Phosphoglycerate mutase	2pg[c] ↔ 3pg[c]	Glycolysis/ Gluconeogenesis

**Table 5:** Reaction showing the largest flux increase after CINADH inhibition. Continuation.

Absolute flux shift mmol gcel^−1^ h^−1^	Reaction name	Reaction formula	Related metabolism
0,0494	Phosphoglycerate kinase	3pg[c] + atp[c] ↔ adp[c] + 13dpg[c]	Glycolysis/ Gluconeogenesis
0,0494	Glyceraldehyde-3-phosphate dehydrogenase	nad[c] + pi[c] + g3p[c] ↔ h[c] + nadh[c] + 13dpg[c]	Glycolysis/ Gluconeogenesis
0,0494	Enolase	2pg[c] ↔ h2o[c] + pep[c]	Glycolysis/ Gluconeogenesis
0,0352	Malonyl-CoA-ACP transacylase	malcoa[m] + ACP[m] ↔ coa[m] + malACP[m]	Fatty acid Biosynthesis
0,0352	Acetyl-Coa carboxylase mitochondrial	accoa[m] + atp[m] + hco3[m] ↔ h[m] + adp[m] + malcoa[m] + pi[m]	Pyruvate Metabolism
0,0211	Triose-phosphate isomerase	dhap[c] ↔ g3p[c]	Glycolysis/ Gluconeogenesis
0,021	Phosphofructokinase	atp[c] + f6p[c] → h[c] + adp[c] + fdp[c]	Glycolysis/ Gluconeogenesis
0,021	Fructose-bisphosphate aldolase	fdp[c] ↔ dhap[c] + g3p[c]	Glycolysis/ Gluconeogenesis
0,0195	Alcohol dehydrogenase (ethanol)	nad[c] + etoh[c] ↔ h[c] + nadh[c] + acald[c]	Glycolysis/ Gluconeogenesis
0,0181	Catalase	2 h2o2[c] → 2 h2o[c] + o2[c]	Free radical Metabolism

## Discussion

### Phenotypic phase planes analysis

The effect of the Available Reducing Capacity (ARC) on ethanol production rate was studied *in-silico* in *S. stipitis* and *S. cerevisiae*. Flux Balance Analysis (FBA) was used to characterize reachable metabolic states in both yeasts at different glucose and oxygen uptake rates. These states are represented as a Phenotypic Phase Plane (PhPP). Metabolic states, ranging from aerobic growth with no ethanol production to metabolic states with low oxygen availability and high ethanol production, using xylose as carbon source were determined (Fig 1).

### Sensitivity analysis

To obtain the sensitivity of ethanol production to changes in ARC, the PhPP was calculated using a bi-level optimization approach which considers biomass and ethanol production as objectives of a nested problem (see methods section). Based on the obtained results, three regions were identified for *S. stipitis*; one in which ARC is in excess, another where ARC has no effect on ethanol production, and a third where ARC is limiting the ethanol production (Fig 2). Whereas for *S. cerevisiae* ARC is always limiting, meaning that an increase in ARC should always lead to an increase in ethanol production (Fig 3). Therefore, the sensitivity analysis indicated that *S. stipitis* and *S. cerevisiae* can be differentiated by their response of ethanol production to ARC, where *S. stipitis* shows a metabolic response to ARC which is less favorable for ethanol production than *S. cerevisiae*. The optimality line for ethanol yield is in the edge of the region between *λ* > 0 and *λ* = 0. In a similar way, Duarte et al, [48] have previously reported that lambda may be used to characterize how the reducing capacity supplied by NADH determines the production of ethanol and acetate in *S. cerevisiae*. Cofactor sensitivities associated to the PhPP have been previously reported in the literature [43, 44], although these works focus only on the sensitivities for biomass production. On the other hand, other reports have used a bi-level optimization approach with the purpose of seeking for metabolic modifications leading to metabolite production improvement [58–60]. Hence, the analysis done in this work achieves a more comprehensive characterization of the metabolic states associated to the PhPP, by establishing a relationship between ARC and the production rate of a metabolite, i.e. ethanol.

### Inhibition of the respiratory chain to improve ethanol production

Reaction deletions reported by Acevedo *et al*, [46] aiming to improve ethanol production in *S. stipitis*, were analyzed using PhPP and sensitivity analysis. The resulting PhPP and its corresponding sensitivities showed that partial inhibition of the reactions reported by Acevedo *et al*, [46] generated a suitable metabolic response in *S. stipitis* for ethanol production. Consequently, inhibitory essays in batch cultures were done in order to verify the effect of inhibiting the respiratory chain. Values for *k*_*L*_*a* of 25 h^−1^ and 15 h^−1^ were considered, which are equivalent to OTR values of 5.9 and 3.5 mmol O_2_ L^−1^⋅h^−1^, respectively. These microaerobic conditions allowed to reach yields between 0.16 g⋅g^−1^ and 0.32 g⋅g^−1^. [61] suggested an OTR range between 0.55 and 1.17 mmol O_2_ L^−1^⋅h^−1^ to reach a yield of 0.29 g⋅g^−1^. On the other hand, [23] reported an OTR of 1.8 mmol O_2_ L^−1^⋅h^−1^ as optimal to reach a yield of 0.40 g⋅g^−1^. [28] suggested an OTR range between 2.5 and 4.7 mmol O_2_ L^−1^⋅h^−1^ to achieve a yield of 0.39 g⋅g^−1^. In the present work rotenone addition allowed to increase yield from 0.16 g⋅g^−1^ to 0.20 g⋅g^−1^ at an OTR of 5.857 mmol O_2_ L^−1^⋅h^−1^ and from 0.32 g⋅g^−1^ to 0.38 g⋅g^−1^ at an OTR of 3.514 mmol O_2_ L^−1^⋅h^−1^. Although yields were not higher than those reported by other authors, increasing ethanol production through respiratory inhibition can be considered as a complementary strategy to aeration control. [62] reported that rotenone addition in batch cultures of *S. stipitis* generates an increment of 40% in ethanol specific productivity and a decrease of 33% in maximum specific growth rate, final yields were not reported. Also, [63] studied the effect of rotenone on xylose fermentation in *S. stipitis*, they reported an increment of 10% in final ethanol concentration.

### Phenotypic phases associated to the uptake rates of the batch cultures

Trajectories over the PhPP were obtained using the uptake rates calculated from the kinetic model, it was observed that the values for *μ* and q_*etoh*_ over the PhPP were close to the ones obtain from the cultures (Figs 7 and 9). Also, it was found that the ethanol sensitivities associated to the trajectories allow predicting an increase in ethanol production to an increase in ARC (*λ* ≤ 0 in Fig 9b). This agrees with the positive effect observed on ethanol yield when inhibiting the Complex I by rotenone addition (see Table 2). Furthermore, when the inhibition of complex I was simulated, the sensitivities showed that ARC was in excess for biomass production, but it was still limiting for ethanol production (see Fig 10). This suggests that increased ethanol production via rotenone addition involves a change in ARC which decreases biomass production, while it has a positive effect on ethanol production.

## Conclusion

In this work sensitivity analysis of genome-scale metabolic modeling and phenotypic phase plane analysis were used to characterize metabolic response on a range of uptake rates in *S. stipitis*, to evaluate the effect of available reducing capacity on ethanol production. The inhibition of NADH dehydrogenase complex I with rotenone leads to an increase in ethanol production in batch cultures and it can be characterized by means of the PhPP and sensitivity analysis of the *S. stipitis* genome scale metabolic network. The analysis shows that cofactor availability, measured as the sensitivity to available reducing capacity $\lambda_{ARC}^{bio}$ can be related with the ethanol yield.
